# Supplementary material for: Overemphasized role of preceding strong El Niño in generating multi-year La Niña events
Source: Nat Commun. 2023 Oct 25;14:6790. doi: 10.1038/s41467-023-42373-5 (PMC10600212; doi:10.1038/s41467-023-42373-5)
Supplement: Supplementary file 1 — Supplementary Information [file 41467_2023_42373_MOESM1_ESM.pdf]

Supplementary Information for:

Overemphasized role of preceding strong El Niño in generating multi-year La Niña events

Ji-Won Kim[1*], Jin-Yi Yu[2*], and Baijun Tian[1]

[1]Jet Propulsion Laboratory, California Institute of Technology, Pasadena, CA, USA

[2]Department of Earth System Science, University of California, Irvine, CA, USA

*Corresponding authors: Ji-Won Kim (jiwon.kim@jpl.nasa.gov); Jin-Yi Yu (jyyu@uci.edu)

This Supplementary Information file includes:

- **Supplementary Texts 1 to 2**
- **Supplementary Tables 1 to 2**
- **Supplementary Figures 1 to 12**
- **Supplementary References**

Supplementary Text 1 | Relationship between a positive PMM and multi-year El Niño events

The relationship between a positive PMM and multi-year El Niño events have been explored in recent studies by Ding et al.[1] and Kim and Yu[2]. These studies specifically investigated how a positive PMM contributes to the occurrence of multi-year El Niño events, utilizing both observations and climate model simulations. The summary of their findings is as follows:

(i) Ding et al.[1] identified the positive phase of the North Pacific Oscillation (NPO) as a crucial factor in the generation of multi-year El Niño events. The NPO signifies an intrinsic extratropical atmospheric variability, manifesting as a north-south seesaw in wintertime sea level pressure anomalies over the North Pacific. During winter, the positive NPO forcing effectively weakens the northeasterly trade winds and warms the SSTs in the subtropical northeastern Pacific. This, in turn, triggers a positive PMM during the succeeding spring. This positive PMM activates the wind-evaporation-SST feedback mechanism, leading to the extension of warm SST anomalies equatorward into the central equatorial Pacific. As a result, a Central Pacific (CP)-type El Niño event develops during the subsequent winter. This CP-type El Niño can excite atmospheric teleconnections to the extratropics, re-energizing the NPO variability and inducing another episode of positive NPO. This cyclic process re-activates the positive PMM and re-triggers the development of another El Niño, resulting in a multi-year El Niño event.

(ii) Kim and Yu[2] also underscored the role of positive PMM as a crucial factor for the generation of a multi-year El Niño event. While the underlying physical mechanisms are broadly akin to those delineated in Ding et al.[1], their emphasis laid on the importance of PMM-involved intra-basin interactions within the same Pacific basin between tropics and subtropics, rather than solely focusing on the role of NPO variability.

It is important to highlight that the subtropical ENSO dynamics associated with the PMM exhibit a more pronounced El Niño-La Niña asymmetry compared to the conventional tropical ENSO dynamics linked to the recharge-discharge processes[3,4]. Consequently, the

conclusions presented by aforementioned studies concerning the positive PMM and multi-year El Niño may not necessarily extrapolate to the negative PMM and multi-year La Niña. Studies like our current research, which explore whether these mechanisms operate differently in the context of positive/negative PMM and multi-year El Niño/La Niña, hold significant value and merit recognition for their novelty.

Supplementary Text 2 | Analysis with the 2200-year-long CESM1 simulation

To address the sampling issue resulting from limited observational period from 1900 to 2022 in this study, we conducted analyses using a 2200-year long-integrated model simulation generated by the Community Earth System Model, version 1 (CESM1). A detailed description of CESM1 is provided by Kay et al.[5], and its simulation outputs are accessible via the Earth System Grid at https://www.cesm.ucar.edu/community-projects/lens/data-sets. The CESM1 has been extensively utilized in ENSO studies since it produces one of the most realistic simulations of the ENSO phenomenon among global climate models by precisely reproducing the observed spatiotemporal characteristics of ENSO[2,6-8]. In the CESM1 analysis, as shown in Supplementary Table 2, we identified 182 multi-year and 49 single-year La Niña events. These events were approximately 17 times larger for multi-year events and 8 times larger for single-year events compared to those observed. Out of the 182 multi-year La Niña events in CESM1, 71 were classified as multi-year La Niña events with a preceding strong El Niño (also referred to as myLN_wPrSEN), and 111 were identified as events without (also referred to as myLN_w/oPrSEN). The remaining 152 La Niña events that did not fit into either the multi-year or single-year category were classified as "neither" events. The methods used to identify and classify La Niña events are the same as those in the observations, except for the use of ±0.5 standard deviations (±0.57°C) instead of ±0.5°C (see 'Identifying La Niña events and their classification in Methods).

Using the events obtained from the CESM1 simulation, we reproduced Figs. 1 to 4, as outlined in the manuscript. The corresponding figures are presented here as Supplementary Figs. 8 to 11. Albeit with some minor discrepancies, such as slightly more westward-extended La Niña structures, the simulation results strongly support the findings of this study, revealing the following:

(i) Supplementary Fig. 8: The excessively emphasized role of preceding strong El Niño in generating multi-year La Niña events (refer to Sect. 'Role of preceding strong El Niño: Overemphasized' in the manuscript)

(ii) Supplementary Fig. 9: The crucial role of negative PMM in generating multi-year La Niña events (refer to Sect. 'Role of negative PMM: Crucial' in the manuscript)

(iii) Supplementary Fig. 10: The physical mechanism responsible for the occurrence of a negative PMM (refer to Sect. ‘Role of negative PMM: Crucial’ in the manuscript)

(iv) Supplementary Fig. 11: The applicability of the PMM mechanism for the generation of triple-dip La Niña events (refer to Sect. ‘Application for triple-dip La Niña’ in the manuscript).

In summary, the results from a long-term CESM1 simulation, offering significantly larger sample sizes, ensure the robustness of the observational findings. Nonetheless, we acknowledge that presenting results from a single climate model simulation may not be sufficient to establish the full robustness. Therefore, future studies should encompass multiple climate model simulations (e.g., CMIP6 model simulations)[9] to provide comprehensive and meaningful conclusions.

Supplementary Table 1 | Statistics of the occurrence frequency of multi-year La Niña events for two distinct periods: ‘Pre-1990’ (spanning from 1900 to 1989, a total of 90 years) and ‘Post-1990’ (spanning from 1990 to 2022, a total of 33 years). The numbers of multi-year La Niña events are derived from Table 1. A notable increase of approximately 55% is observed during the Post-1990 period in comparison to the Pre-1990 period.

**Period**	**Pre-1990 (1900–1989)**	**Post-1990 (1990–2022)**
Number of multi-year La Niña events	7	4
Events per decade	0.78	1.21

Supplementary Table 2 | Numbers of La Niña events classified in the CESM1 simulation and the observations. Numbers in parentheses in the 'Multi-year La Niña' column denote the occurrences of triple-dip La Niña events.

Number of events	Multi-year La Niña	w/ a preceding strong El Niño	w/o a preceding strong El Niño	Single-year La Niña	Neither La Niña
CESM1	182 (40)	71	111	49	152
OBS	11 (5)	4	7	6	5

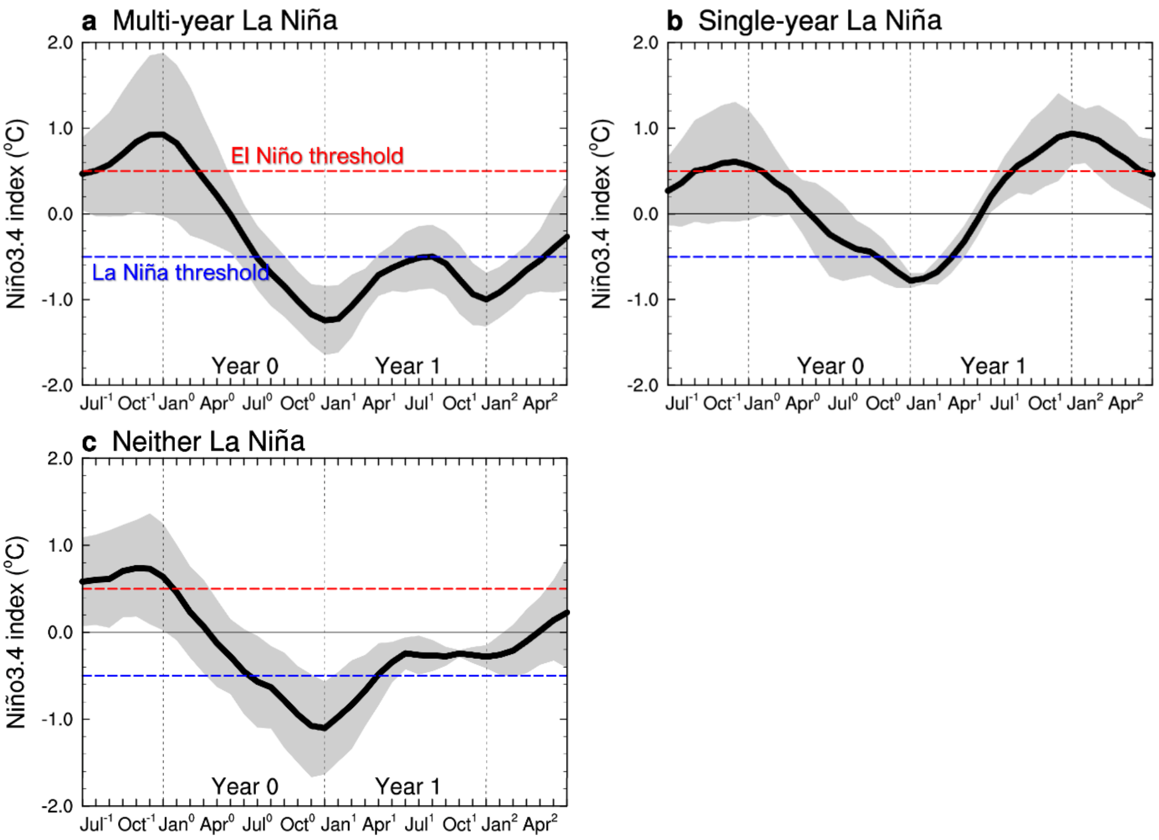

Supplementary Fig. 1 | Evolution characteristics of the multi-year, single-year, and neither La Niña events. a–c, Composite evolution of Niño3.4 index for the multi-year (**a**), single-year (**b**), and neither (**c**) La Niña events. The index is smoothed with a three-month running-mean filter and the shaded areas represents its ±1 s.d. Red and blue dashed lines indicate thresholds for El Niño (0.5°C) and La Niña (−0.5°C), respectively.

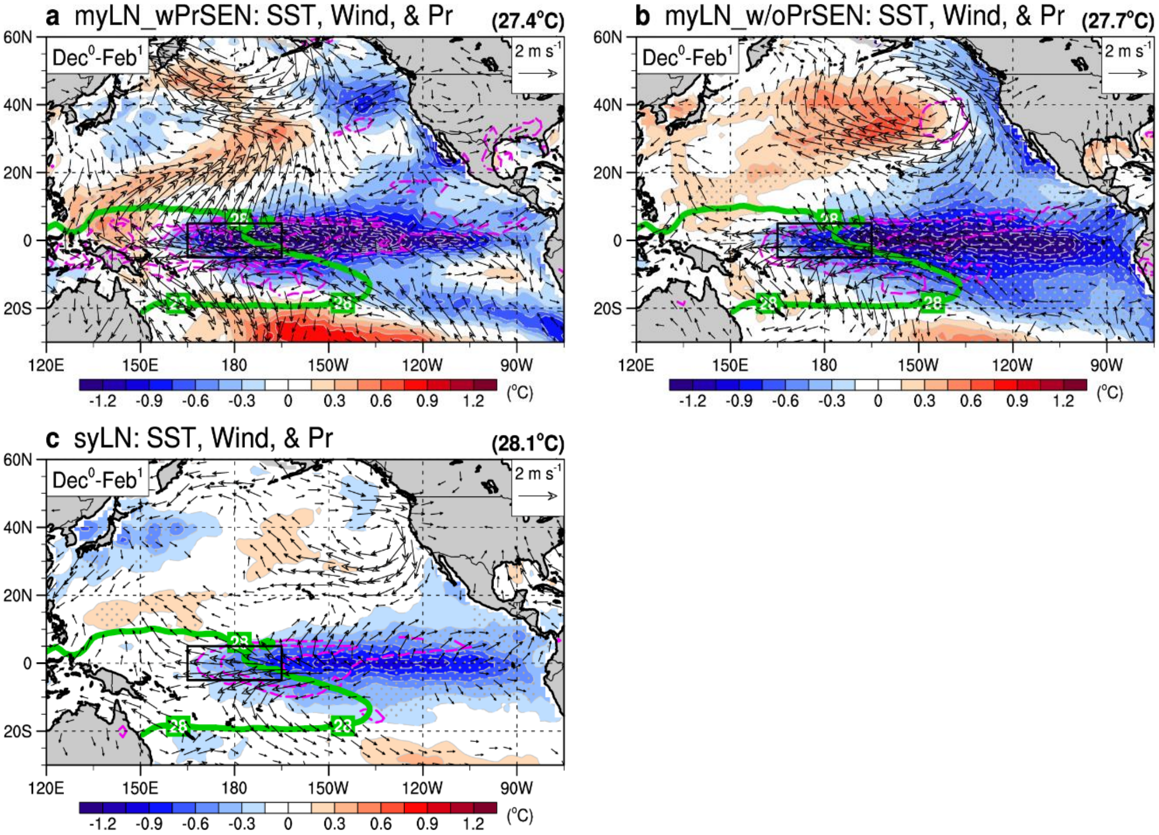

Supplementary Fig. 2 | Oceanic and atmospheric structures during the winter of the first La Niña. a–c, Composite structures of anomalous sea surface temperature (SST; shading, in °C), surface wind (vector with minimum intensity > 0.4 m s^{-1}), and precipitation (Pr; represented by magenta dashed contour lines with a contour interval of 1 mm d^{-1}, and negative values only expressed) during the first winter for myLN_wPrSEN (**a**), myLN_w/oPrSEN (**b**), and syLN (**c**). The green contours represent the climatological 28°C isotherm line, which depicts an area for the western Pacific warm pool. Gray stippling indicates regions where SST anomalies are statistically significant based on the Student's *t*-test. The raw SSTs averaged over the tropical central Pacific (5°S–5°N, 165°E–165°W; black rectangle) are marked in the top right corner of the panels.

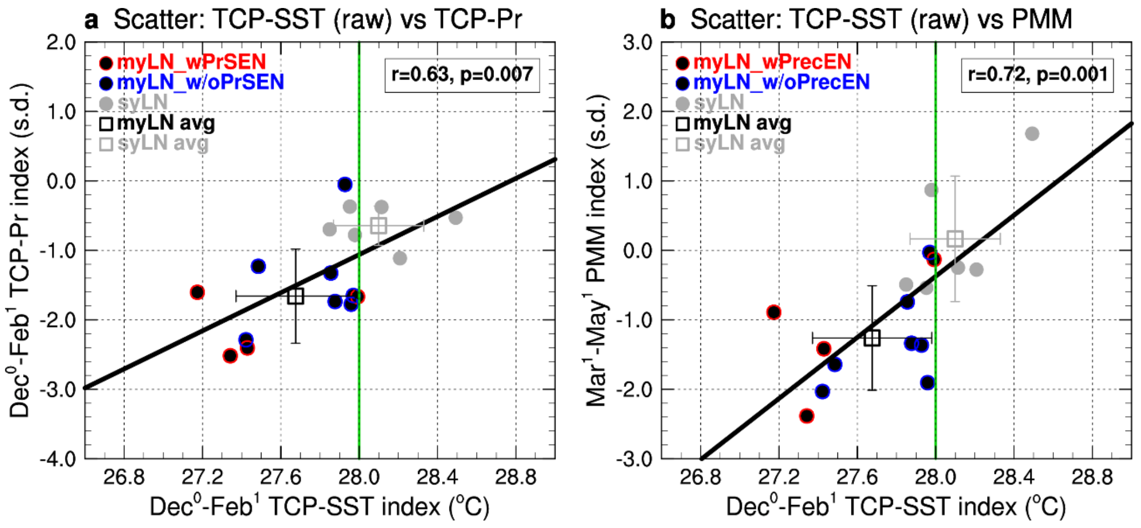

Supplementary Fig. 3 | Relationships between raw sea surface temperature (SST) in the tropical central Pacific and in-situ precipitation (Pr) anomalies in the first winter, as well as the subsequent spring PMM. a, Scatter plot of TCP-SST index values against TCP-Pr index values during the first winter for myLN_wPrSEN (black dots with red outline), myLN_w/oPrSEN (black dots with blue outline), and syLN (gray dots) (see 'Definition of climate indices' in Methods). The black (gray) square represents the average for multi (single)-year La Niña events with error bars indicating ±1 s.d. Correlation coefficient (r) between the two indices and its p-value (p) are marked in the upper right corner. The green line indicates 28°C, which is the convective threshold temperature over the tropics. **b**, Same as **a**, except using PMM index during the subsequent spring. Notably, multi-year La Niña events, regardless of their group, exhibit TCP-SST index values below the threshold temperature, along with strong positive correlations for both TCP-Pr (r=0.63) and PMM (r=0.72) index values.

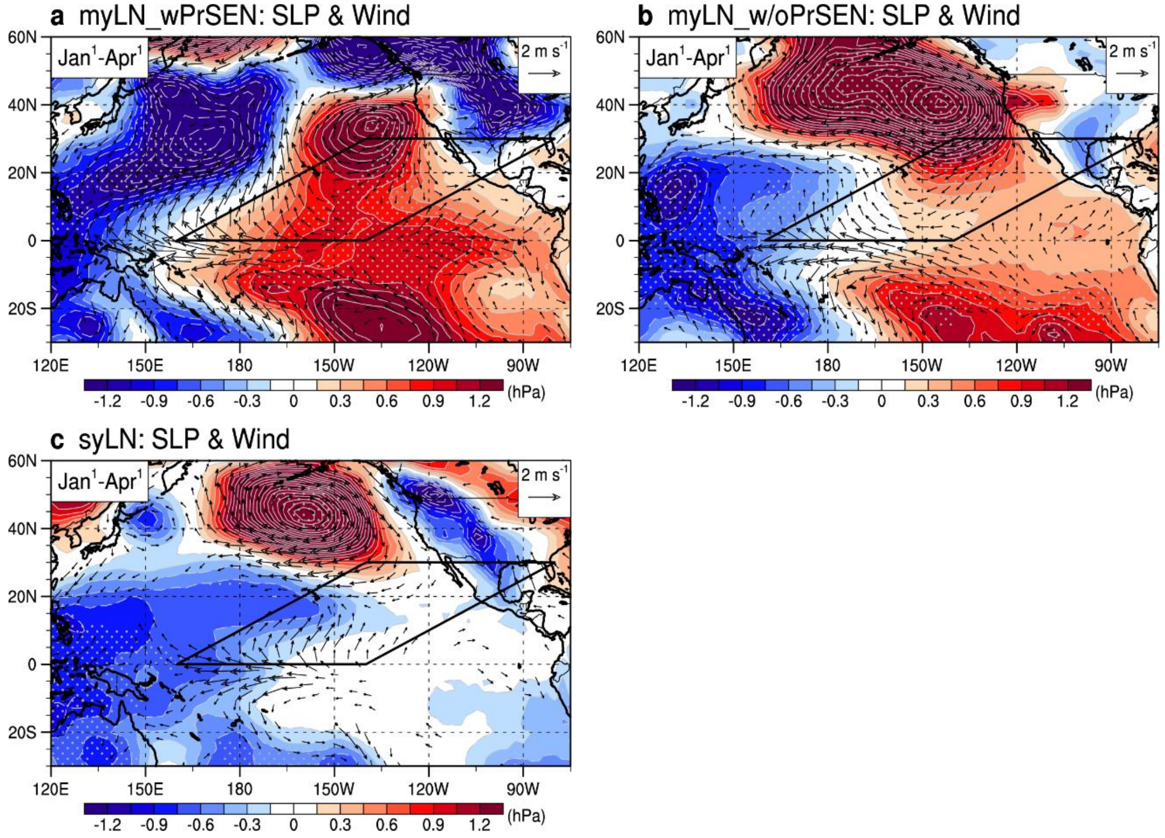

Supplementary Fig. 4 | Atmospheric structures during the transition season between the first winter and spring. Composite structures of anomalous sea level pressure (SLP; shading, in hPa) and surface wind (vector with minimum intensity > 0.4 m s^{-1}) during the first winter for myLN_wPrSEN (**a**), myLN_w/oPrSEN (**b**), and syLN (**c**). The black parallelograms delineate the region where a negative PMM occurs and exerts its influence. Gray stippling indicates regions where SLP anomalies are statistically significant based on the Student's *t*-test.

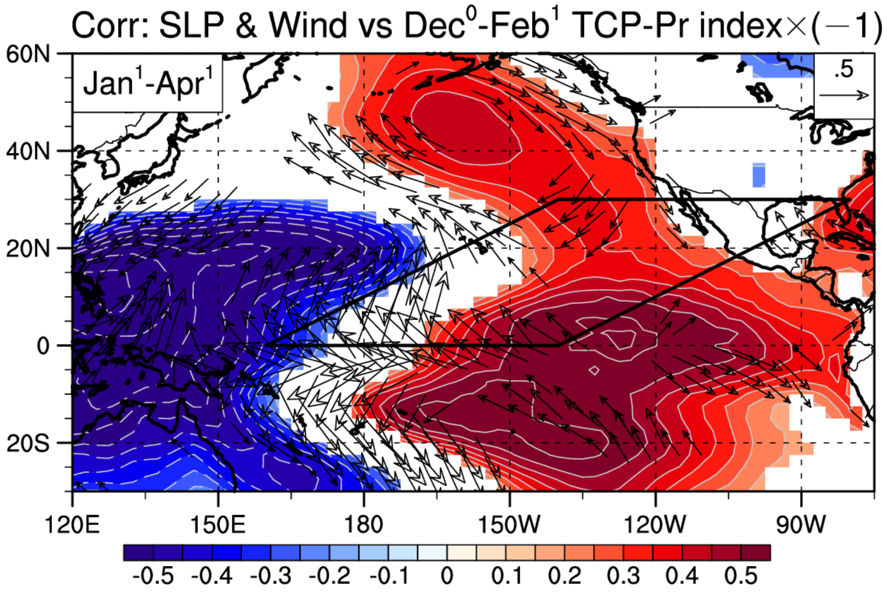

Supplementary Fig. 5 | Causality between the tropical central Pacific suppression and the subtropical North Pacific anticyclone anomaly. Lagged correlation map of anomalous sea level pressure (SLP; shading) and surface wind (vector) during January^1 to April^1 against the TCP-Pr index during December^0 to February^1 for the period 1900–2022. Only areas displaying significant correlation coefficients at the 95% confidence level based on the Student's *t*-test are shown. The TCP-Pr index is multiplied by −1, so that its positive values correspond to negative diabatic heating anomalies. The black parallelogram delineates the region where a negative PMM occurs and exerts its influence.

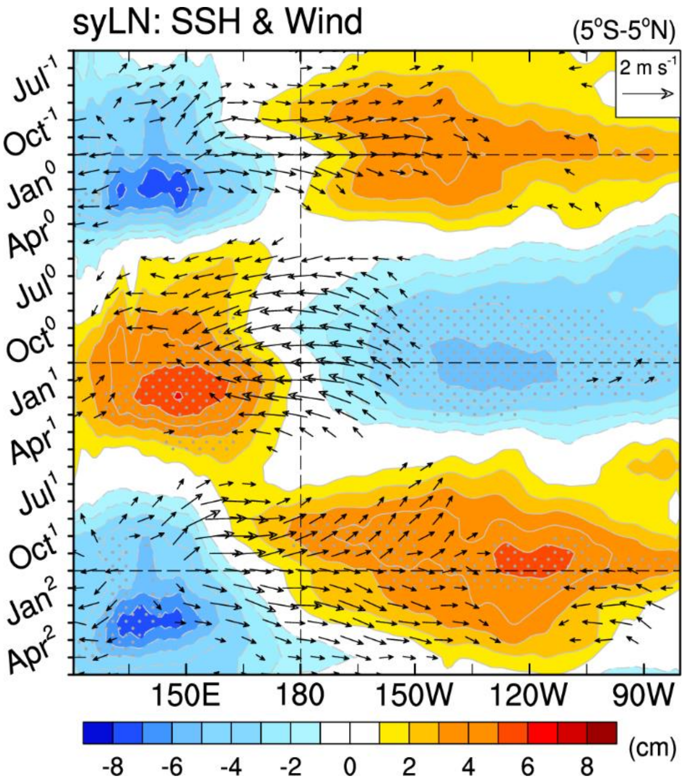

Supplementary Fig. 6 | Spatiotemporal evolution of the equatorial Pacific heat content during single-year La Niña. Longitude-time plots of equatorial (5°S–5°N) Pacific anomalous sea surface height (SSH; shading, in cm) and surface wind (vector with minimum intensity > 0.4 m s^{-1}) for syLN. Gray stippling indicates regions where SSH anomalies are statistically significant based on the Student's *t*-test.

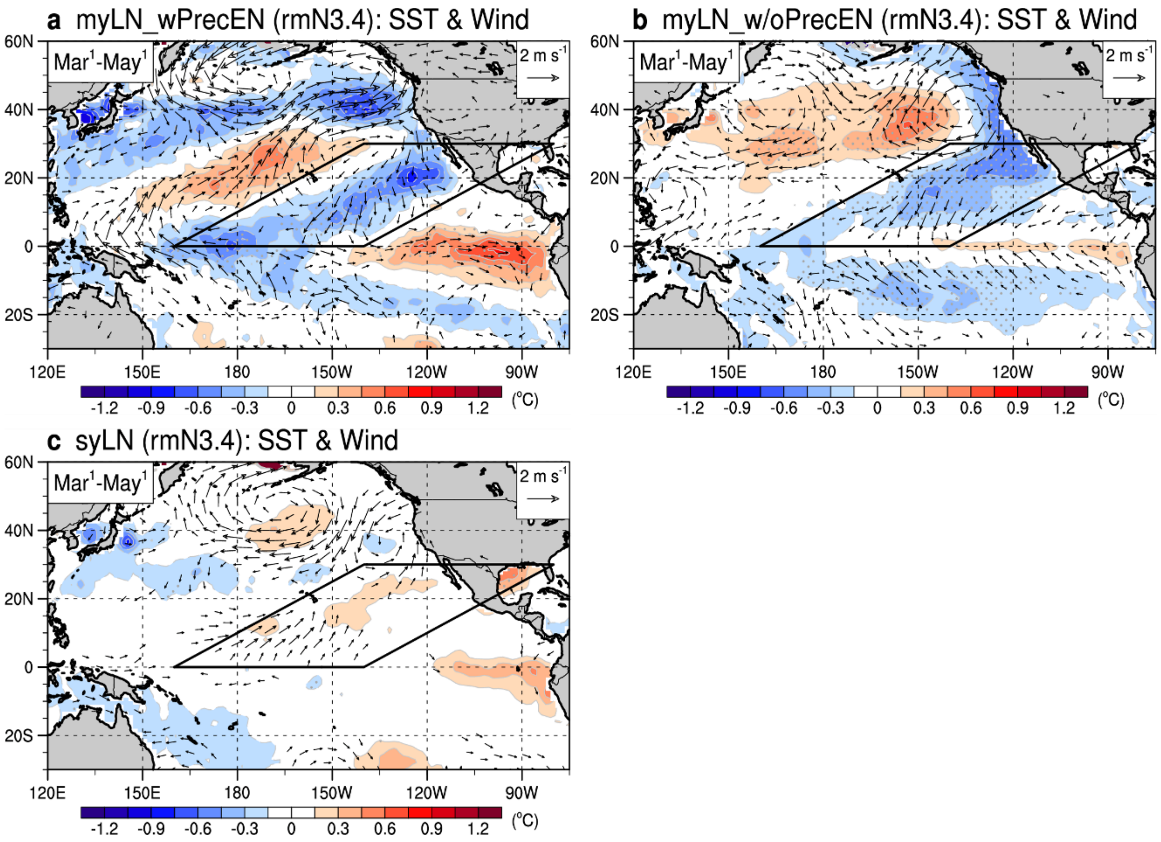

Supplementary Fig. 7 | Oceanic and atmospheric structures during the second spring, with ENSO signals removed. Same as Fig. 2a-c in the manuscript except with ENSO signals removed by regressing out the Niño3.4 index from sea surface temperature (SST) and surface wind fields prior to analysis.

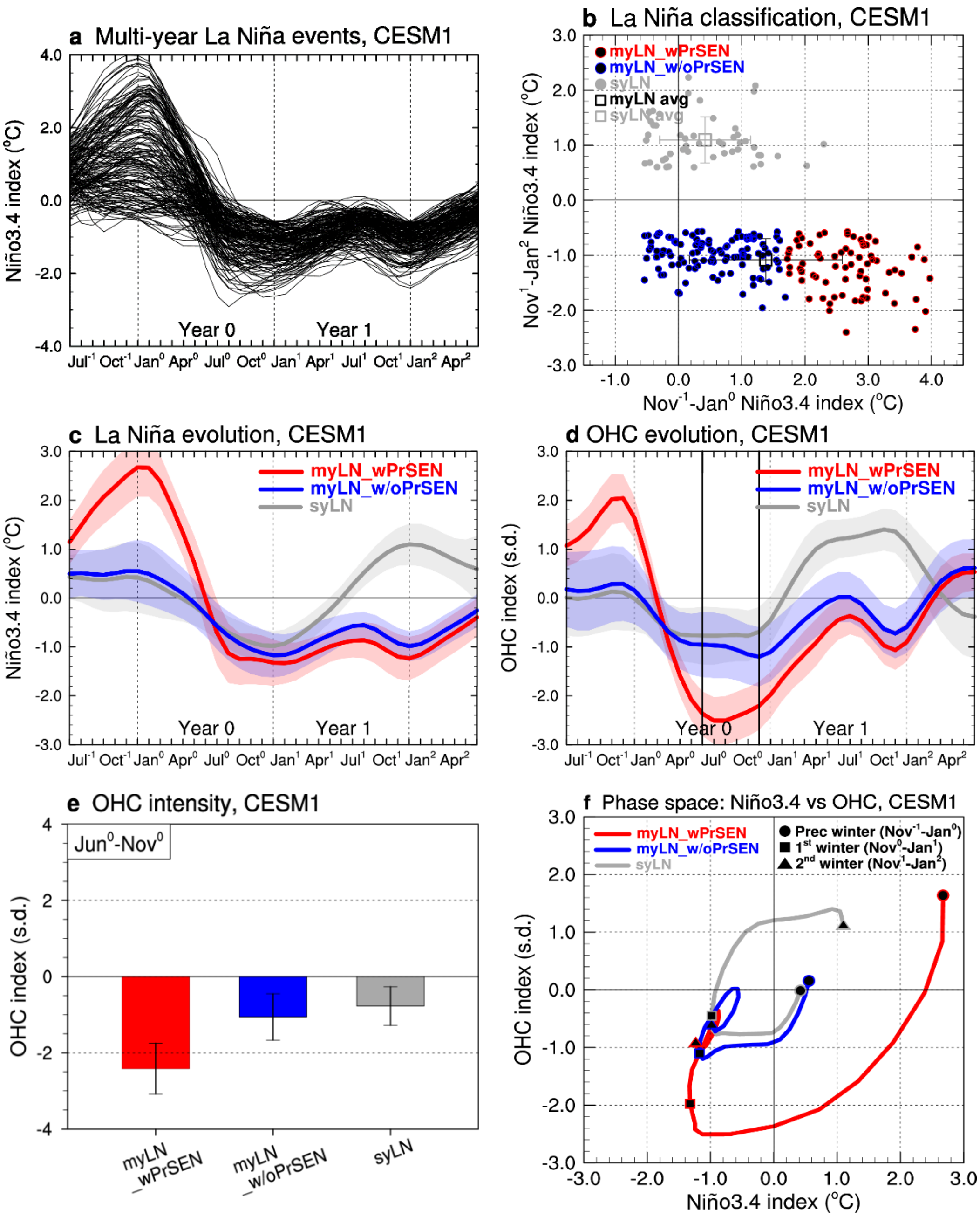

Supplementary Fig. 8 | The excessively emphasized role of preceding strong El Niño in generating multi-year La Niña events. Same as Fig. 1 in the manuscript except using the CESM1 simulation

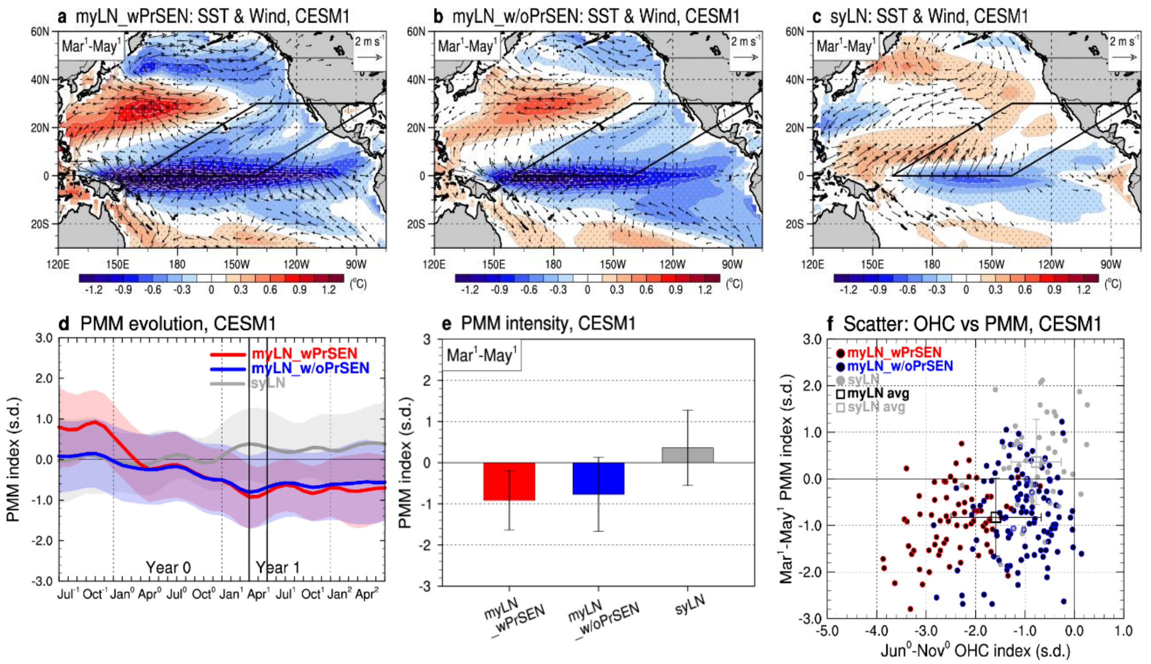

Supplementary Fig. 9 | The crucial role of negative PMM in generating multi-year La Niña events. Same as Fig. 2 in the manuscript except using the CESM1 simulation

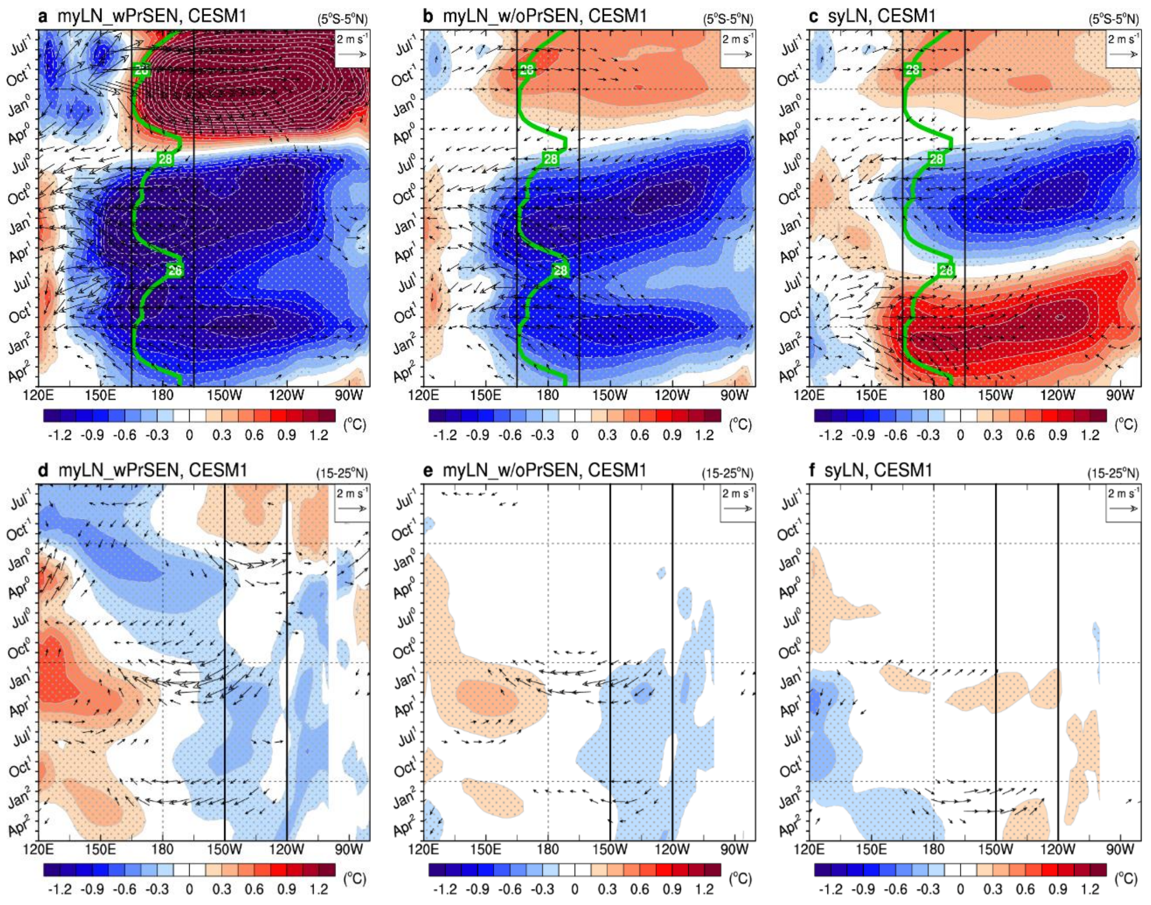

Supplementary Fig. 10 | The physical mechanism responsible for the occurrence of a negative PMM. Same as Fig. 3 in the manuscript except using the CESM1 simulation

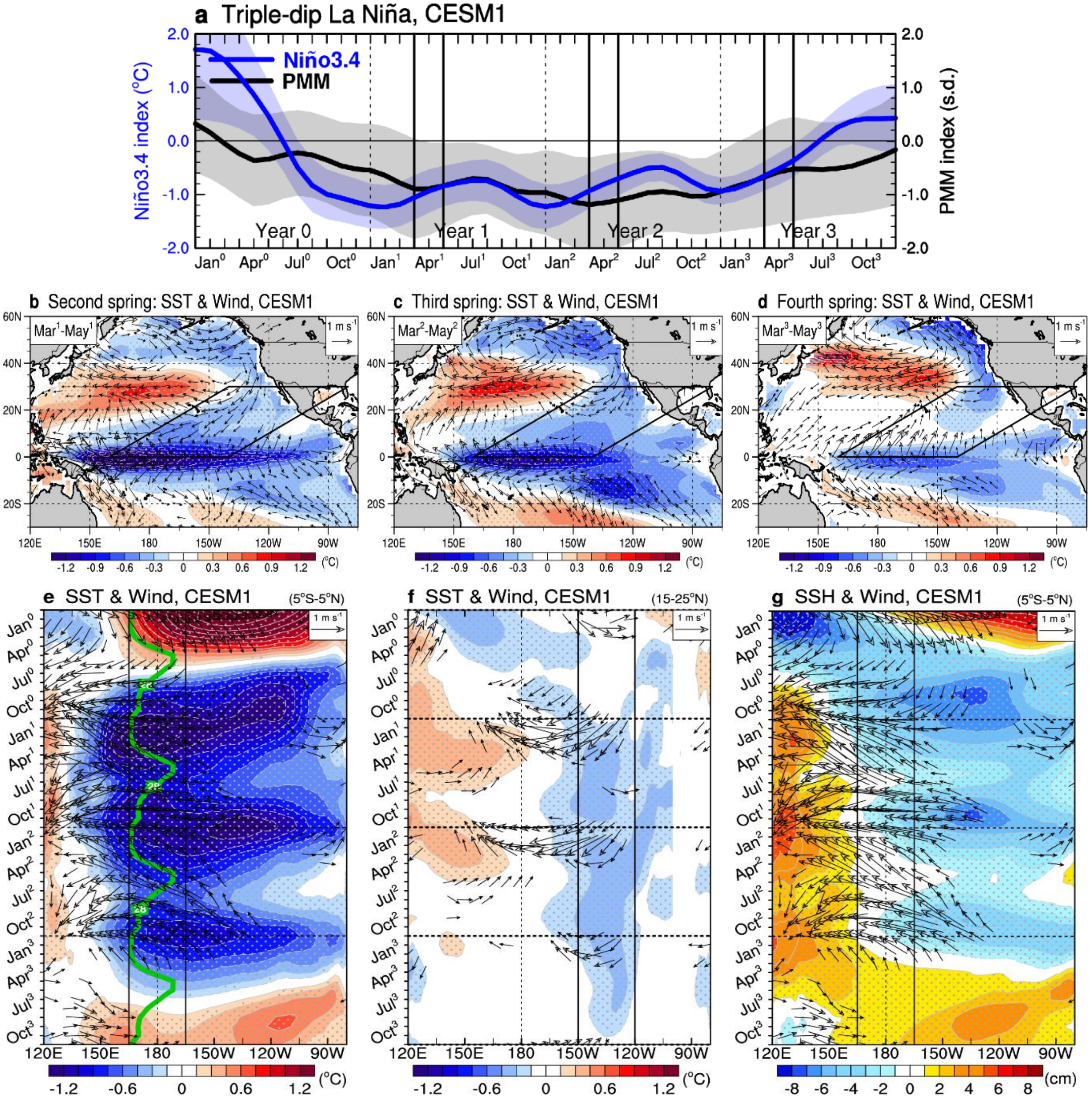

Supplementary Fig. 11 | The applicability of the PMM mechanism for the generation of triple-dip La Niña events. Same as Fig. 4 in the manuscript except using the CESM1 simulation

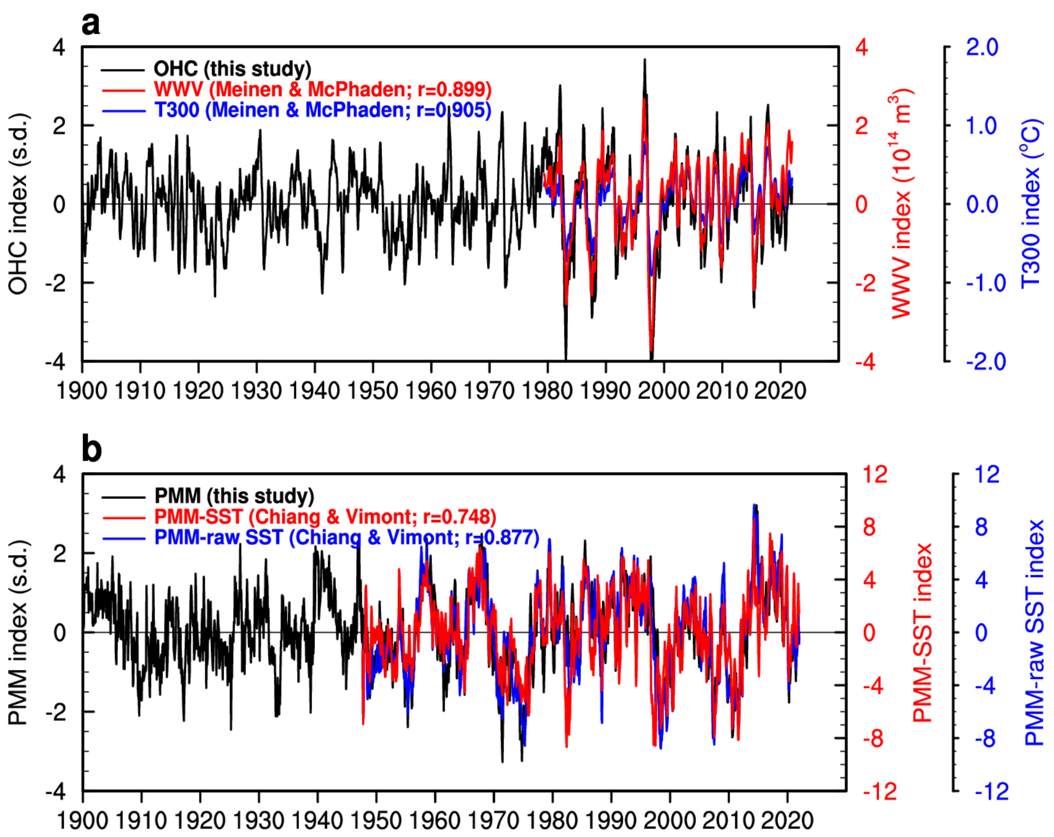

Supplementary Fig. 12 | Reliability of OHC and PMM indices used in this study. a, Index evolutions of OHC (this study, black curve), warm water volume (WWV, red curve), depth averaged temperature in the upper 300 meters (T300, blue curve). Meinen and McPhaden[10] originally developed the WWV and T300 indices, and the data can be accessed at https://www.pmel.noaa.gov/elNiño/upper-ocean-heat-content-and-enso. **b**, Same as **a**, except using PMM index (this study, black curve), PMM-SST index (red curve), and PMM-raw SST index (blue curve). Chiang and Vimont[11] originally suggested the PMM-SST and PMM-raw SST indices, and the data can be obtained at https://www.aos.wisc.edu/~dvimont/MModes/Data.html. The correlation coefficients (r) with OHC index over 1980–2022 (in panel **a**) and PMM index over 1948–2022 (in panel **b**) are marked.

Supplementary References

1. Ding, R., Tseng, Y. H., Di Lorenzo, E., Shi, L., Li, J., Yu, J. Y., ... & Li, F. (2022). Multi-year El Niño events tied to the North Pacific Oscillation. *Nature communications*, *13*(1), 3871.

2. Kim, J. W., & Yu, J. Y. (2022). Single-and multi-year ENSO events controlled by pantropical climate interactions. *npj Climate and Atmospheric Science*, *5*(1), 88.

3. Yu, J. Y., & Fang, S. W. (2018). The distinct contributions of the seasonal footprinting and charged-discharged mechanisms to ENSO complexity. *Geophysical Research Letters*, *45*(13), 6611-6618.

4. Fan, H., Wang, C., & Yang, S. (2023). Asymmetry between positive and negative phases of the Pacific Meridional Mode: A contributor to ENSO transition complexity. *Geophysical Research Letters*, *50*(14), e2023GL104000.

5. Kay, J. E., Deser, C., Phillips, A., Mai, A., Hannay, C., Strand, G., ... & Vertenstein, M. (2015). The Community Earth System Model (CESM) large ensemble project: A community resource for studying climate change in the presence of internal climate variability. *Bulletin of the American Meteorological Society*, *96*(8), 1333-1349.

6. DiNezio, P. N., Deser, C., Okumura, Y., & Karspeck, A. (2017). Predictability of 2-year La Niña events in a coupled general circulation model. *Climate dynamics*, *49*(11-12), 4237-4261.

7. Wu, X., Okumura, Y. M., & DiNezio, P. N. (2019). What Controls the Duration of El Niño and La Niña Events?. *Journal of Climate*, *32*(18), 5941-5965.

8. Zhu, T., & Yu, J. Y. (2022). A Shifting Tripolar Pattern of Antarctic Sea Ice Concentration Anomalies During Multi-Year La Niña Events. *Geophysical Research Letters*, *49*(23), e2022GL101217.

9. Eyring, V., Bony, S., Meehl, G. A., Senior, C. A., Stevens, B., Stouffer, R. J., & Taylor, K. E. (2016). Overview of the Coupled Model Intercomparison Project Phase 6 (CMIP6) experimental design and organization. *Geoscientific Model Development*, *9*(5), 1937-1958.

10. Meinen, C. S., & McPhaden, M. J. (2000). Observations of warm water volume changes in the equatorial Pacific and their relationship to El Niño and La Niña. *Journal of Climate*, *13*(20), 3551-3559.

11. Chiang, J. C., & Vimont, D. J. (2004). Analogous Pacific and Atlantic meridional modes of tropical atmosphere–ocean variability. *Journal of Climate*, *17*(21), 4143-4158.
